# A case of diverse psychiatric and functional impairments following immune checkpoint inhibitor therapy

**DOI:** 10.1002/pcn5.70297

**Published:** 2026-02-05

**Authors:** Takako Ikegami, Ryoichi Sadahiro, Junji Yamaguchi, Eri Nishikawa, Saho Wada, Tatsuto Terada, Rika Nakahara, Hiromichi Matsuoka

**Affiliations:** ^1^ Department of Palliative Medicine National Cancer Center Hospital Chuoku Tokyo Japan; ^2^ Cancer Chemotherapy Center Osaka Medical and Pharmaceutical University Takatsuki City Osaka Japan; ^3^ Department of Psycho‐Oncology National Cancer Center Hospital Chuoku Tokyo Japan; ^4^ Department of Supportive and Palliative Care Development Center National Cancer Center Hospital Chuoku Tokyo Japan

**Keywords:** central nervous system immune‐related adverse events, gastric cancer, immune checkpoint inhibitor, immune‐mediated encephalitis, psycho‐oncology

## Abstract

**Background:**

Immune checkpoint inhibitors (ICIs) can cause a range of immune‐related adverse events (irAEs), including rare neuropsychiatric complications. However, these events often present with diverse and non‐specific symptoms, making diagnosis difficult.

**Case Presentation:**

A 64‐year‐old man with Stage IV gastric cancer receiving nivolumab developed impaired consciousness, delusions, and dissociative behavior, and reduced instrumental activities of daily living during and after ICI therapy. During chemotherapy, the patient was suspected of having dementia and was referred to a psychiatrist. However, the possibility of irAE was not mentioned at that time. Despite normal magnetic resonance imaging (MRI) and cerebrospinal fluid findings, a multidisciplinary assessment due to clinical features and exclusion of other etiologies led to the clinical suspicion of an immune‐related encephalopathy. Steroid pulse therapy and antipsychotics (risperidone, later olanzapine) improved symptoms. Psychiatric relapse occurred after discontinuing risperidone and resolved with olanzapine.

**Conclusion:**

In patients undergoing ICI therapy, new‐onset psychiatric symptoms should raise suspicion for irAEs. Timely multidisciplinary intervention is essential for accurate diagnosis and effective symptom management.

## INTRODUCTION

Immune checkpoint inhibitors (ICIs) have been used since 2014 to treat various solid tumors and lymphomas. In gastric cancer, nivolumab combined with chemotherapy has demonstrated promising results (response rate: 57%, disease control rate: 71.8%).^1^ However, serious immune‐related adverse events (irAEs) remain a concern. Among them, immune‐mediated encephalitis is rare (<1% incidence) but potentially fatal, with a reported mortality rate of 19%.^2^ The condition presents with a wide range of symptoms—altered mental status (55%), cognitive impairment (52%), seizures (29%), psychiatric disturbances (16%), and autonomic dysfunction (33%)^3^—making timely diagnosis and management particularly difficult. This report presents a case of a patient who developed complex psychiatric symptoms and impaired consciousness during ICI therapy. The case illustrates the diagnostic challenges and therapeutic considerations in managing such neuropsychiatric symptoms during ICI treatment. Although neurological irAEs caused by ICIs have been increasingly reported, most cases involve clear neurological manifestations such as seizures or impaired consciousness. In contrast, cases in which psychiatric symptoms—such as delusions, dissociative behavior, or impulsivity— remain rare but under‐recognized. Moreover, few reports have described detailed therapeutic courses in which both immunosuppressive and antipsychotic treatments contributed to recovery. This case highlights ICI‐related neuropsychiatric toxicity and underscores the importance of collaboration between oncologists and psychiatrists.

## CASE DESCRIPTION

A 64‐year‐old man presented with impaired consciousness, speech and behavioral disturbances, monologuing, incontinence, and difficulty communicating.

### Life history

He smoked 20 cigarettes/day for 9 years and drank 1000 mL of beer or 300 mL of wine daily. He graduated from college and worked full‐time as a physician, remaining at work even after his gastric cancer diagnosis. There is no family history of mental illness or epilepsy.

### Clinical course

In March 2023, the patient began to experience upper abdominal discomfort and pain. He subsequently consulted his home doctor, who diagnosed him with anemia (Hb 6.9 g/dL). In early April of the same year, a magnetic resonance imaging (MRI) scan showed no malignant tumor. Later that month, he was diagnosed with gastric cancer (cStage IV: T4aN1M1) and started neoadjuvant chemotherapy with TS‐1, oxaliplatin, and nivolumab in May 2023. Nivolumab was administered at a dose of 360 mg per cycle for a total of three cycles (total 1080 mg). During chemotherapy, the first episode of loss of consciousness occurred on his way home after the third administration of nivolumab, leading to a self‐inflicted traffic accident. Within 1 month, he caused a second traffic accident; in this episode, he drove to a dealer with the hood up, showing impulsive and reckless behavior. Around the same time, he also began to have episodes where he would lose the ability to talk, his personality changed to become aggressive, verbally abuse his eldest daughter, who was a key person and with whom he had a good relationship, eat while unconscious, and not be able to name his medications, even though he was a physician. Although his family confirmed that these psychiatric and behavioral episodes emerged after the third nivolumab administration, the exact onset dates could not be reliably determined, as neither the patient nor the family could recall day‐level details. According to collateral history from his daughter, there was no apparent change in his alcohol consumption before the onset of psychiatric symptoms. Suspecting dementia, the family visited the psychiatrist to evaluate his cognitive function, but no abnormalities were found, and the possibility of irAE encephalitis was not mentioned by his psychiatrist. According to his family and his preserved functioning as an actively practicing physician, no clinically significant cognitive impairment was apparent before the onset of psychiatric symptoms. Also, these behaviors were not reported to his oncologist.

In July, he underwent a pyloric gastrectomy. Three days later, he developed delirium followed by a seizure. He was transferred to another hospital for a neurological consultation as encephalitis was suspected. He exhibited inappropriate behaviors, incontinence, and delusions about medical costs. Psychotic symptoms included hypochondriacal, guilt, and poverty delusions, believing his property was confiscated. Worsening seizures required intubation and 3000 mg levetiracetam. Differential diagnoses included irAE encephalitis, epilepsy after nivolumab, other autoimmune or paraneoplastic encephalitis, depression, and delirium.

After transfer to another hospital, a head MRI and cerebrospinal fluid (CSF) examination were performed, but no abnormal findings were found. Electroencephalogram performed twice showed no epileptiform discharges, and no findings suggesting diffuse slowing were reported. The CSF also ruled out infection. Depression was ruled out as the patient did not meet the diagnostic criteria, lacking persistent depressed mood or loss of interest. Schizophrenia and paranoid disorder were also ruled out based on clinical presentation. As autoantibody tests, including anti‐Hu, Ma2, and NMDA antibodies, were not performed due to a lack of insurance coverage in Japan, autoimmune encephalitis could not be excluded. Based on treatment history, an ICI‐induced neuropsychiatric adverse event was suspected. Steroid pulse therapy improved communication, but persistent delusions required levetiracetam tapering and 0.5 mg risperidone. Delusions subsided in a week, and he was discharged seizure‐free. Later, he returned for a gastric cancer follow‐up. After learning of rising tumor markers, he had sudden speech and behavioral changes, monologues, and incontinence, leading to an urgent psycho‐oncology referral.

**Figure 1:**
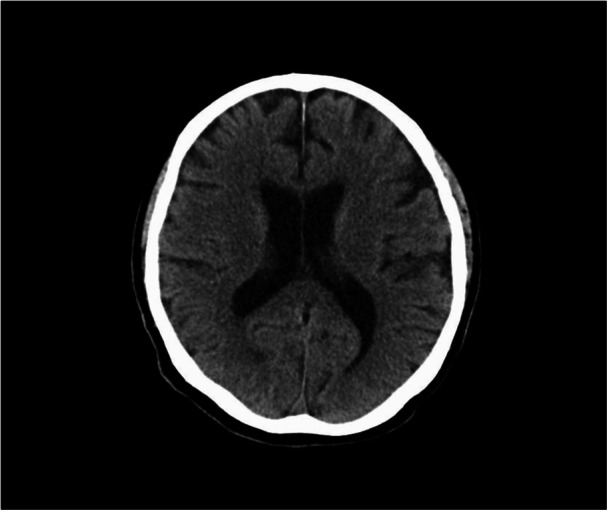
Head computed tomography (CT) on the day of consultation to the psycho‐oncology department, showing no brain metastasis, hemorrhage, and hydrocephalus.

**Figure 2:**
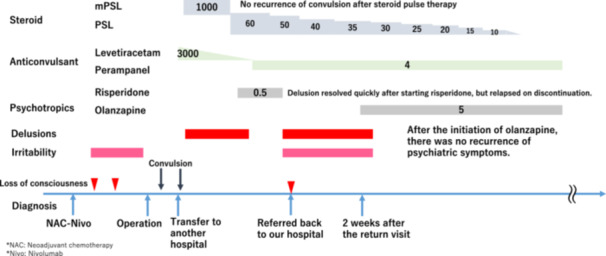
Clinical course of the patient. mPSL, methylprednisolone; PSL, prednisolone.

The head computed tomography (CT) was performed for further examination, after which the level of consciousness improved. At the first consultation at the Department of Psycho‐Oncology, the patient maintained eye contact, but his reactions were delayed. His verbal output was reduced, but communication was still possible. His attention and cognitive abilities were reduced, but there were no impairments, and there were no signs of paralysis or other neurological abnormalities. A CT scan of the head (Figure 1) showed no lesions or brain atrophy. The episode of sudden unconsciousness following the emotional impact of elevated tumor markers, along with his rapid recovery, was clinically interpreted as a psychogenic disturbance of consciousness, possibly dissociative in nature. This was considered to be associated with stress vulnerability, potentially exacerbated by prior neuropsychiatric effects of ICI therapy. His mental disorder, predominantly manifested as delusional beliefs, had exhibited notable improvement following the administration of steroid pulses and risperidone (0.5 mg/day, oral) during his previous hospitalization. However, he had self‐disrupted risperidone after discharge from his previous hospital, which may have precipitated a relapse of his mental disorder. At this time, the administration of steroids, prednisolone (PSL) 50 mg/day, was still ongoing. Although his communication improved over time, his daughter reported his abnormal nocturnal behaviors that he woke up and ate snacks unconsciously in the middle of the night. He was also experiencing symptoms of emotional instability, insomnia, and delusions. However, he refused to take the medication at the first consultation, despite attempts to resume the antipsychotic medication. At a return visit two weeks later, he continued to exhibit symptoms of psychosis, including delusions of guilt (e.g., “the police are after me”) and paranoia (e.g., “I cannot receive nivolumab because of my daughter”). Additionally, he reported insomnia, sudden expressions of anger and violent outbursts, and emotional lability. He remained emotionally unstable, and his anger toward his daughter, who was a central figure in his life and lived with him, was impeding his familial relationships in his daily life. Furthermore, he showed impaired reality judgment, evident in ordering and ingesting overseas medication without verification, despite being a physician and attempting to work despite reduced instrumental activities of daily living (IADLs) like driving and medication management. Given the patient's pronounced irritability, olanzapine 5 mg was initiated to leverage its mood‐stabilizing and antipsychotic effects. Following the initiation of olanzapine therapy, the patient's delusional and irritable symptoms rapidly abated, his family relationship with his daughter was restored, and he gradually returned to work as a physician. PSL, which had been initiated based on a clinical suspicion of irAE encephalitis, was gradually reduced from a dosage of 50 to 7 mg over a period of 2 months. In January 2024, the patient was continued on olanzapine 5 mg and discontinued PSL, yet there was no recurrence of psychiatric symptoms. In February, he was diagnosed with recurrent gastric cancer, and postoperative chemotherapy with S‐1 was reinitiated. No relapse of psychiatric or behavioral symptoms occurred during this period. Due to the distance between his residence and our hospital, he was referred to a local physician in May (Figure 2).

## DISCUSSION

This case illustrates the diagnostic challenges posed by neuropsychiatric symptoms following ICI therapy. Recurrent mental disorders with impaired consciousness impacted daily life, safety, and relationships, including traffic accidents and deteriorating relationships. IrAEs can affect any organ, with CTCAE Grade 3 or higher events occurring in 22%–24%^4^ of ipilimumab monotherapy, 5%–10%^5^, ^6^ with nivolumab or pembrolizumab, and 55%^7^ with combination therapy. Neurological irAEs are seen in approximately 4% of cases,^8^ with encephalitis/myelitis and meningitis being particularly rare (0.84% and 0.36%, respectively).^2^, ^9^ The exact mechanism of psychiatric symptoms in irAE encephalitis remains unclear, but a recent study suggests that morphological activation and major histocompatibility complex (MHC) Class II upregulation on microglia by ICI is associated with neurological irAEs.^10^ Typical forms include autoimmune limbic encephalitis affecting the hippocampus and temporal lobe, with diverse symptoms such as headache, fever, altered consciousness, and hallucinations. Some cases test positive for autoantibodies like anti‐Ma2, anti‐Hu, and anti‐NMDA,^11^ suggesting that mechanisms include cellular immunity and NMDA receptor dysfunction.^12^ Previous literature has often focused on ICI‐associated encephalitis presenting with neurological findings, making it difficult to recognize cases dominated by psychiatric symptoms as irAE‐related manifestations and complicating differential diagnosis from psychiatric disorders. Diagnosis is made through MRI and CSF analysis, though it can be challenging due to the absence of definitive findings in imaging and laboratory tests.^13^ Cases without definitive findings require clinical judgment. In this case, MRI, CT, and spinal fluid were normal, but irAE encephalitis was suspected due to loss of consciousness after nivolumab administration and exclusion of other causes (central nervous system metastasis or cancer recurrence). Although a formal causality score was not calculated, the clinical course supported a possible association with nivolumab, as notable psychiatric and consciousness‐related episodes were observed after the third administration, and no recurrence occurred when chemotherapy was resumed without nivolumab. The seizures improved with steroids, and antipsychotics managed psychiatric symptoms. Steroid psychosis was ruled out as symptoms preceded steroid use and relapsed without dose changes. Delirium was excluded due to stable symptoms, no physical causes, and seizure presence. Sudden unconsciousness with behavioral and verbal abnormalities after stopping risperidone suggested brain stress vulnerability induced by ICI treatment‐induced neurotoxicity. While psychiatric irAEs have a reported incidence of 2.71%,^14^ effective therapies are not yet established. In this case, steroids and antipsychotics like risperidone and olanzapine were beneficial, indicating their potential in managing irAE‐induced psychiatric symptoms. Improvement with combined corticosteroid and antipsychotic therapy suggests that both immune‐mediated mechanisms and neurotransmitter dysregulation may contribute to the pathophysiology. Early recognition and treatment of irAE should be considered in patients receiving ICIs who present with altered consciousness and psychiatric symptoms.

## CONCLUSION

When psychiatric symptoms arise in patients undergoing ICI therapy, promptly consider irAEs. Psychiatric symptom–dominant irAEs are rare but likely under‐recognized, and timely collaboration between oncology and psychiatry teams is crucial for accurate diagnosis and effective management.

## AUTHOR CONTRIBUTIONS

Takako Ikegami and Ryoichi Sadahiro contributed to the conception and design of the study, the collection and analysis and interpretation of the research data, and the writing of the manuscript and critical revision of its intellectual content. Junji Yamaguchi and Hiromichi Matsuoka contributed to the interpretation of the research data and critical revision of the important intellectual content of the manuscript. All authors consented to the final approval of the submitted paper and published manuscript, as well as the accountability of the study.

## CONFLICT OF INTEREST STATEMENT

The authors declare no conflicts of interest.

## ETHICS APPROVAL STATEMENT

Ethics approval was obtained from the institutional review board of the National Cancer Center Hospital.

## PATIENT CONSENT STATEMENT

Written informed consent for publication was obtained from the patient.

## CLINICAL TRIAL REGISTRATION

N/A.

## Data Availability

The data that support the findings of this study are available on request from the corresponding author. The data are not publicly available due to privacy or ethical restrictions. All data generated or analyzed during this study are included in this published article.
